# Comparing the Discussion of Telehealth in Two Social Media Platforms: Social Listening Analysis

**DOI:** 10.1089/tmr.2023.0008

**Published:** 2023-08-03

**Authors:** Catherine C. Shoults, Leah Dawson, Corey Hayes, Hari Eswaran

**Affiliations:** ^1^ Institute for Digital Health and Innovation, University of Arkansas for Medical Sciences, Little Rock, Arkansas, USA.; ^2^ Department of Biomedical Informatics, University of Arkansas for Medical Sciences, Little Rock, Arkansas, USA.; ^3^ Center for Mental Healthcare and Outcomes Research, Central Arkansas Veterans Affairs Healthcare System, North Little Rock, Arkansas, USA.; ^4^ Department of Obstetrics and Gynecology, University of Arkansas for Medical Sciences, Little Rock, Arkansas, USA.

**Keywords:** social media, data mining, telehealth, telemedicine, social listening, twitter, reddit

## Abstract

**Background:**

Social media is used as a source of information and platform to discuss health care; however, there is little research on discussion of telehealth in social media. Past research has looked at individual platforms, but a comparison of discussion on two platforms (Reddit and Twitter) has not been performed. Understanding telehealth-related social media discourse and the differences between platforms may provide insights into how telehealth is characterized online and which platforms provide patient perspectives. The COVID-19 pandemic provides a unique case study to examine how social media users approached both Reddit and Twitter during an international health crisis. This study used natural language processing tools and two social media platforms to (1) characterize and contrast each platform's telehealth-related posts according to themes and (2) assess the frequency of telehealth and telehealth-related terms posts before and during the onset of the COVID-19 pandemic.

**Methods:**

We collected 6 years (2016 through 2021) of social media posts from Twitter and Reddit. The themes of the corpus were extracted using hashtags, subreddits, and Latent Dirichlet Allocation (LDA) and were analyzed using descriptive statistics.

**Results:**

Both Twitter and Reddit showed exponential growth in the use of the term “telehealth” and telehealth-related terms in early 2020. The use of telehealth-related terms and discussion of COVID-19 coincided in both social media sites; however, other themes were discussed, including how to use telehealth. Reddit LDA clusters showed greatest usage of “telehealth” when associated with using or suggesting telehealth for receiving therapy, counseling, or psychoanalysis while Twitter focused on sharing telehealth news, products, and services.

**Discussion:**

Twitter and Reddit had extensive growth in the use of telehealth-related terms after the COVID-19 pandemic. Twitter and Reddit showed themes connecting COVID-19 to telehealth, especially in reference to services, therapy, and counseling, however, Reddit had more discussion suggesting use of telehealth services or requesting peer insights into how to use telehealth as compared with Twitter, which appeared more focused on telehealth as a business or product.

## Introduction

While the definition of telehealth varies, the United States Department of Health and Human Services defines telehealth as providing care for a patient without an in-person office visit.^1^ Telehealth is most commonly associated with the internet, and the use of synchronized video visits or asynchronous internet-based messaging.^2^ The COVID-19 pandemic highlighted one of the greatest strengths of telehealth; patients and doctors can now interact without being in the same place.^2^

Taking the need for physical proximity out of health care provides additional routes for accessing medical resources and provides an avenue to decrease the spread of infectious diseases. As discussed in their review of telehealth utilization during the COVID-19 pandemic, Garfan et al. note that the pandemic caused a great disruption in health care, including routine outpatient visits being cancelled, a decrease in the delivery of routine medical care, and a shortage of health care resources.^2^ The onset of the pandemic resulted in significant growth of the use of telehealth according to analysis of encounter data.^3^

Social media represents an explosive, disruptive communication and information sharing technology that has changed how people interact on topics from the most mundane meme to paramount life decisions. According to the Pew Research Center 2021 survey results, ∼70% of Americans use social media.^4–7^ Social media data have an outsized impact on health care due to its universal nature, allowing greater communication opportunities for those with health care issues.^7–9^

In the United States, 80% of internet users use the web to search for health information, and a large proportion of these users turn to social media for information.^10,11^ Health care systems and researchers are tapping into social media as a big data tool for mining information and understanding patient perspectives on many health care-related topics.^12–14^

Social media and telehealth became intertwined during the coronavirus disease pandemic (COVID-19). According to a recent COVID-19 and social media scoping review, “Social media became a crucial communication tool for information generation, dissemination, and consumption.”^15^ Because of the transition to telehealth during the COVID-19 pandemic, social media became a communication tool for telehealth.^2^ Further, telehealth-related journal articles have seen explosive growth showing the interconnected nature of a global pandemic and the need for easy-to-access health care outside of clinics and hospitals.^16^ The user-generated content around COVID-19 can provide patient perspectives on their experiences related to telehealth.^17^

Social listening, the technique of using social media user perspectives to learn about their thoughts on a subject, has been previously used in health care.^18^ A few studies have employed social listening to understand how COVID-19 has impacted social media discussion around telehealth; the few studies that have assessed telehealth and social media are limited in time frame or focus entirely on Twitter.^19–21^

Massaad and Cherfan analyzed the geospatial distribution of tweets associated with telehealth during the pandemic but limited the analysis to 1 week at the beginning of the disease spread in the United States.^19^ Champagne-Langabeer et al. used five months of Twitter data focused on telehealth to understand the sentiment of “telehealth” and “telemedicine” posts. Results found that most posts were positive or neutral.^20^

Pollack et al. used over a year of tweets and also found that telehealth-related tweets were associated with positive sentiments.^21^ Anderson et al. used 1 month of data in 2020 and 2021 to compare the social acceptance of the telemedicine industry due to COVID-19. The analysis used only Twitter data and found that the pandemic moved the discussion of telehealth from introduction of a service to widespread adoption.^22^

While we are starting to understand the impact of COVID-19 on telehealth, there is a lack of understanding of the difference between user discussion of telehealth-related terms on different social media platforms. This research is designed to help understand how Reddit and Twitter users are using social media, which may help provide insights into how targeted interventions can use social media to improve health.

Therefore, this study used natural language processing tools and two social media platforms, Twitter and Reddit, to (1) assess the frequency of telehealth and telehealth-related posts before and during the onset of the COVID-19 pandemic and (2) characterize each platform's telehealth-related posts according to theme. Twitter and Reddit were chosen for this study for three reasons.

First, these platforms are used as social media forums, and each can provide insights into user perspectives. Second, Twitter and Reddit provide a form of content tagging—Twitter uses hashtags and Reddit uses sub-forums called “subreddits.” Third, both platforms had robust Application Programming Interfaces (APIs), allowing researchers to access historical posts. Overall, this study aimed at providing novel insight into how telehealth-related terms were discussed in both Twitter and Reddit (i.e., as categorized by themes) and identifying trends in telehealth-related tweets and posts over the course of the COVID-19 pandemic.

## Methods

### Data extraction

Twitter and Reddit data from January 2016 through December 2021 were used for this study. Twitter archives were accessed using the Twitter Academic Developer API. The Academic API allows researchers to access historical tweets. JavaScript Object Notation (JSON) formatted data were downloaded from the Academic API using twarc2 command line to search for specific terms in the Twitter archive.

The twarc2 command included requests to exclude retweets and promoted-only advertisements (null cast). Reddit was accessed via the API Pushshift.io.^23^ Both submissions and comments were downloaded and are collectively called “posts” in this analysis. The search terms for Twitter and Reddit were “Telehealth,” “Telemedicine,” “Remote Health,” “Distance Health,” “Connected Health,” “Mobile Health,” “mHealth,” “Virtual Care,” “Virtual Health,” “Digital Health,” “Electronic Health,” “Internet Medicine,” “eHealth,” and “Cybermedicine.” These terms were chosen after a scoping review was conducted to identify telehealth-related terms. Results from the scoping review were reported separately.^24^

### Data cleaning

**FIG. 1.**
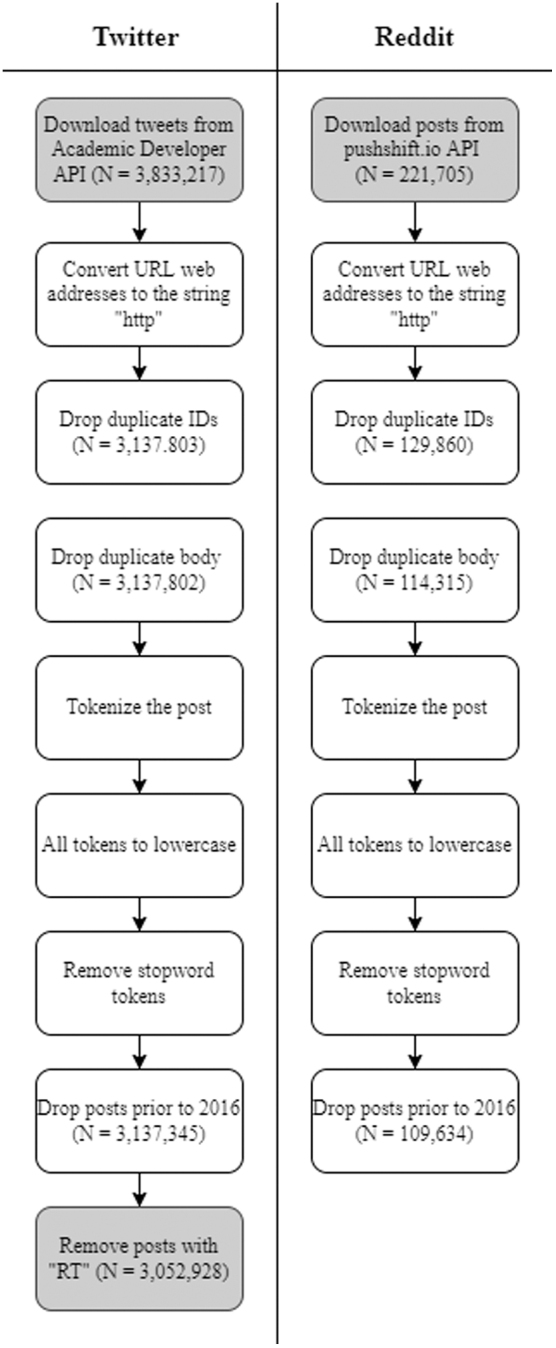
Flowchart of cleaning methods for Twitter and Reddit. *Gray boxes* identify differences in methodology.

Python 3.8.10 was used to access, clean, and analyze data (Fig. 1). Twitter posts (i.e., tweets) were downloaded in. JSONL format, and the twarc2 flattening command was used to create comma separated text files. Reddit files were downloaded as. JSON files, and *Pandas* was used to create data frames, which were saved as comma separated text files.^25^ There are two types of Reddit posts: submissions and comments. Reddit submissions have both a title and body, which were combined into one post. Redditors can post comments under a submission. Comments do not contain a title and did not require additional processing to create a post.

Tweets and Reddit posts were cleaned using natural language processing tools. Specifically, *TweetTokenizer* from the *NLTK*.*tokenize* toolkit was used to separate string text into lists of tokens to maintain hashtags and URL addresses.^26^ All tokens were converted to lowercase, and key phrases had spaces removed to maintain their meaning in one token. Stopwords, low information words, were removed from the list of tokens using *NLTK*.*corpus* stopwords in English.

The URLs of tweets were converted to the string “*http*.” This allowed for de-duplication of posts that had the same text but linked to different URLs. Table 1 shows an example Tweet and Reddit post before and after Natural Language Processing (NLP) cleaning.

**Table 1.** Comparison of Example Synthetic Tweet and Reddit Post Before and After Natural Language Processing Cleaning (Tokenizing, Lowercase, Dropping Stopwords, Rejoining Post)

	Twitter	Reddit
*Original text*	The #Vets #Telemedicine allows physicians to use #telehealth in rural towns https://t.co/6twuersc	I posted about having a child with autism and my wife's Crohns getting worse. She is interested in therapy due to depression. What with Coronavirus do you have any therapist recommendations that offer telehealth?
*Cleaned text*	#vets #telemedicine allows physicians use #telehealth rural towns http	Posted child autism wifes crohns getting worse interested therapy due depression coronavirus therapist recommendations offer telehealth

In the Twitter column, note that the URL was changed to “http” and that all hashtags are maintained.

### Data analysis

Twitter uses hashtags (#) to allow for topic tagging. These tags provide insights into Twitter trends and provide natural self-categorization. As part of the cleaning process, COVID-19-related terms were grouped into the string “covid.” Grouped COVID-19 terms [after applying .lower() to all strings] include the following: “covid-19,” “covid 19,” “sars cov 2,” “sars-cov-2.” Top hashtags were extracted using regex and grouped by search term. After cleaning and de-duplicating the tweets, descriptive statistics were run in Python and visualizations were created in Tableau 2020.2.17.

Reddit posts are tied to a community called a subreddit. While subreddits are a form of classification, there are thousands of subreddits. The large population can limit the ability to understand broad themes. Latent Dirichlet Allocation (LDA) was used to generate a theme for each Reddit and Twitter post. LDA is a probabilistic model that represents posts as a distribution of topics.

The goal of LDA is to infer the topic distributions for each post by generating the most likely word distributions within that post's topic. *Gensim.models.LdaMulticore* package was run with 20 *passes* and *minimum_probability* set to 0, which means that the machine should not filter out any of the generated topics.^27^

The hyperparameter *num_topics* was tested via coherence plots. *Num_topics* tells the machine how many clusters or topics to find in the Reddit posts that is decided by the user. Fine tuning of the number of topics hyperparameters tested numbers in the range from 10 to 100 by increments of 10. The package *pyLDAvis* was used to visualize the results of the LDA model.^28^ When visualizing the results, the parameter lambda was set to zero to see terms that were used often within each theme but not outside of that cluster.

Although LDA supplies topic labels, these labels may focus on words that do not pertain to health or may not be easily interpretable as a theme. To help solve this issue, a t-distributed stochastic neighbor embedding (t-SNE) plot to reduce dimensionality to two dimensions was created to visualize the posts and the clusters were analyzed both in the context of their LDA topic's list of words and by examining example posts or tweets from the cluster. The t-SNE was created using *sklearn manifold TSNE* and visualized with the *Bokeh* library (Bokeh 2.2.3).^29^ The Bokeh packages *io*, *models*, and *palettes* were used to create an interactive scatter plot that allowed for filtering out years and a hover tool that showed the post text and LDA label via custom JavaScript. Analysis was performed on an NVIDIA DGX-1.

In addition to studying the posts with t-SNE plots, the relevance metric of LDA was used to better understand topic clusters. The LDA's relevance metric helps when interpreting LDA results. This metric was created by Sievert and Shirely and is defined as follows^30^:

$$\begin{array}{cc} relevance\left(termw|topict\right) & =\lambda \times p\left(w|t\right)\\ & +\left(1-\lambda \right)\times p\left(w|t\right)∕p\left(w\right) \end{array}$$

When the metric is set to 1, the metric is equal to *p*(*w* | *t*) and topic terms are ranked solely based on their probability of appearing within the topic. This means that common words throughout the corpus may appear in the topic words. When the relevance metric is set to 0, the metric is equal to *p*(*w* | *t*)/*p*(*w*) so terms are ranked based on how much more probable the word is to be found within the topic as compared with the entire corpus. The relevance metric at 0 terms may help distinguish from other topics. The default for LDA is to supply topic labels that use the relevance metric at 1.

## Results

The results include the analysis of 3,052,928 tweets and 109,634 Reddit posts, which will be referred to as social interactions. Table 2 breaks down the annual total number and percentage of telehealth-related social interactions. During the years 2016–2019, our analysis reported a decline of telehealth-related tweets, ranging from 15% to 11.8%, respectively. While our analysis showed an increase in Reddit posts over those same years, the percentage range was 1.9–6.5%, significantly less than that of Twitter.

**Table 2.** (Twitter) Counts of Tweets Related to “telehealth” or “telehealth” Related Terms by Years Including the Percent of Total Number of Tweets Over Time

Year	Twitter No. of tweets (percent of tweets)	Reddit No. of posts (percent of posts)
2016	457,761 (15.0)	2130 (1.9)
2017	390,630 (12.8)	3053 (2.8)
2018	396,663 (13.0)	5056 (4.6)
2019	359,943 (11.8)	7173 (6.5)
2020	847,767 (27.8)	50,548 (38.0)
2021	600,164 (19.7)	41,674 (46.1)

(Reddit) Counts of posts related to “telehealth” or “telehealth” related terms by years including percent of total number of posts over time.

**FIG. 2.**
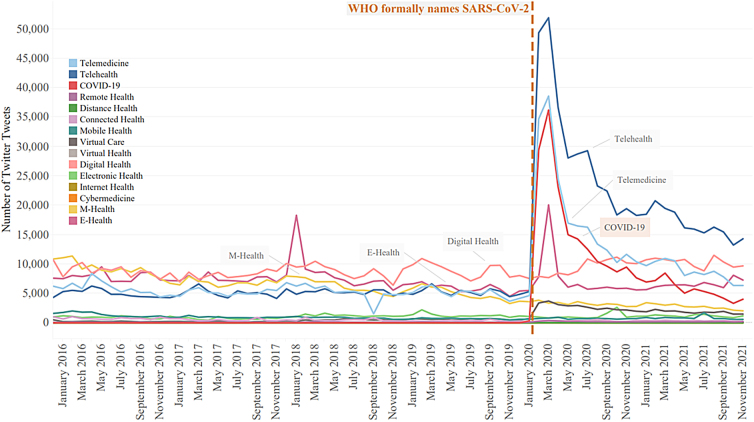
Count of telehealth-related tweets containing telehealth-related terminology from January 2016 through December 2021. The *orange*, *vertical dashed line* represents WHO naming of the SARS-CoV-2 disease on February 11, 2020. Twitter used the terms “m-health,” “e-health,” and “digital health” consistently before the pandemic. After COVID-19, the terms “telehealth” and “telemedicine” spiked in growth and maintained high use over time.

**FIG. 3.**
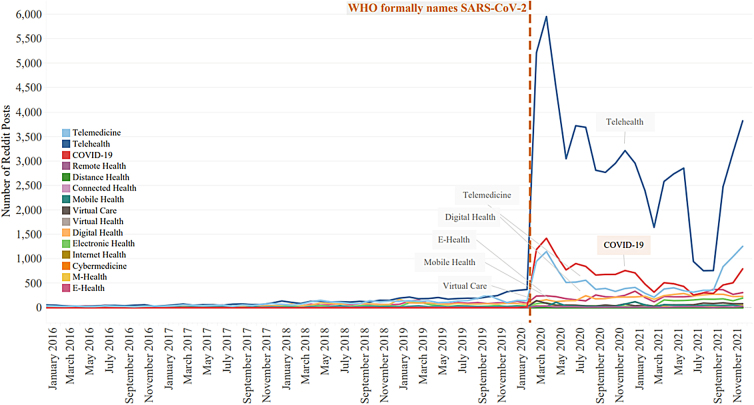
Count of telehealth and telehealth-term-related Reddit posts (submissions and comments) from January 2016 through December2021. The *orange*, *vertical dashed line* represents WHO naming of the SARS-CoV-2 disease on February 11, 2020. Reddit post counts remained low and stable until the onset of the coronavirus pandemic in the United States, which led to growth in February through April 2020 and then decline in total count through July 2021. September 2021 showed a renewed growth in telehealth and telehealth-related terms.

While total Twitter posts remained consistently higher than Reddit posts, the total number of social interactions containing telehealth-related terms remained somewhat stable in both platforms during the years 2016–2019. Tweets during those years fluctuated by ∼98,000 tweets, and Reddit posts fluctuated by 5043 posts. During the 2020 coronavirus pandemic, tweets jumped from around 350,000 tweets in 2019 mentioning “telehealth” terms to 850,000 tweets in 2020. Figure 2 shows this growth with the dotted orange line marking the formal naming of SARS-CoV-2 by the World Health Organization. Reddit showed the same increase in February 2020 (Fig. 3). As compared with Twitter's leveling-out of the number of tweets, Reddit showed renewed growth in telehealth-term posts starting in September of 2021.

Table 3 provides the distribution of the top 10 Reddit subreddits and Twitter hashtags for “telehealth” and “telemedicine” from January 2016 through December 2021. Twitter's use of hashtags helps sort the topics that the Twitter user deems applicable to their tweet. These hashtags can be used to sort tweets. Reddit does not use hashtags and does not have an equivalent tagging function; however, Reddit posts are submitted to topic-labeled communities called “subreddits.”

**Table 3.** Distribution of the Top 10 Reddit Subreddits and Twitter Hashtags for “telehealth” and “telemedicine” Containing Posts/Tweets over the Timeframe January 2016 Through December 2021

Reddit top subreddits (r/)		Twitter top hashtags (#)	
Telehealth top 10 subreddit (# posts)	Telemedicine top 10 subreddits (# posts)	Telehealth top 10 hashtags (# tweets)	Telemedicine top 10 hashtags (# tweets)
Psychotherapy (1794)	Healthcare (326)	#telehealth (332,272)	#telemedicine (249,919)
TalkTherapy (1565)	TeleMedicine (303)	#telemedicine (74,928)	#telehealth (73,164)
AskDocs (1157)	AutoNewspaper (292)	#healthcare (61,228)	#healthcare (48,304)
Advice (1247)	Suboxone (289)	#digitalhealth (44,005)	#digitalhealth (28,714)
AskReddit (970)	Testosterone (252)	#covid19 (42,730)	#covid19 (27,942)
ADHD (1069)	Medicine (224)	#healthtech (18,774)	#mhealth (14,414)
Coronavirus (737)	ADHD (176)	#health (17,836)	#health (13,918)
wallstreetbets (654)	Health (148)	#mhealth (17,828)	#healthtech (13,037)
ABA (824)	*Name withheld* (136)	#healthit (17,828)	#ehealth (9741)
Therapy (474)	wallstreetbets (120)	#mentalhealth (13,929)	#healthit (8341)

Note that “Name withheld” represents a Reddit username and is not reported to protect their privacy.

The analysis of the telehealth-related search terms showed that Reddit posts discussed the term “telehealth” more frequently than any other term. That term was reported in 62% of the telehealth-related posts, followed by the term “telemedicine” reported in 15% of the telehealth-related posts. Reddit posts are submitted to online communities, known as subreddits. The topics of discussion in these subreddits for “telehealth” include therapy, advice, and world-markets/investments, with the most posts being in the “Psychotherapy” community.

The topics of discussion in the subreddits for “telemedicine” focus on health care topics, with the top subreddit “Healthcare” focusing on insurance. The other topics of discussion include telemedicine, telehealth, technology interventions in health care, English-language Really Simple Syndication (RSS) feeds, and specific health topics including suboxone, testosterone, and Attention Deficit Hyperactivity Disorder (ADHD). An individual Reddit user who focuses on blockchain protocol for telehealth was listed as a top 10 subreddits for “telemedicine” because the term #telemedicine is repeated on every post of the user's profile page; however, as this was a Redditor's username, it was not included in Table 3 to maintain their privacy.

The most commonly used Twitter hashtags for “telehealth” and “telemedicine” from January 2016 through December 2021 included #telehealth with 332,272 tweets and #telemedicine with 249,919 tweets. Other top hashtags included during this timeframe were #healthcare, #digitalhealth, #covid19, #healthtech, #mhealth, #health, #healhit, #ehealth, and #mentalhealth.

Before the start of the pandemic, Twitter had constant use of the terms “m-health,” “e-health,” and “digital health.” In February 2020, telehealth-related tweets spiked when COVID-19 was declared a disease by the World Health Organization. While the term “telemedicine” showed an increase, the term “telehealth” exhibited a greater increase. In April 2020, the #covid19 hashtag quickly dropped from its high while the #telehealth hashtag declined and began to plateau.

Reddit posts showed a spike in “telehealth” and other telehealth-related terms from January 2016 to December 2021. Before the onset of the pandemic, Reddit had little use of any telehealth-related terms. The term “telehealth” was the most commonly used term in this corpus, and unlike Twitter, telehealth-related posts appeared to show a renewed growth in September 2021.

**FIG. 4.**
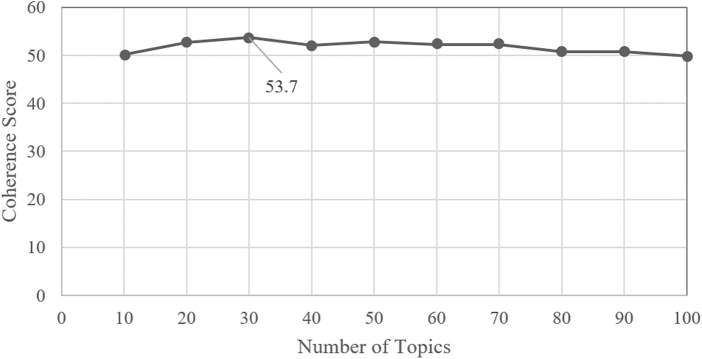
Coherence plot to determine optimal number of topics for Reddit LDA topic modeling. Thirty topics had the highest coherence score (53.7). LDA, Latent Dirichlet Allocation.

**FIG. 5.**
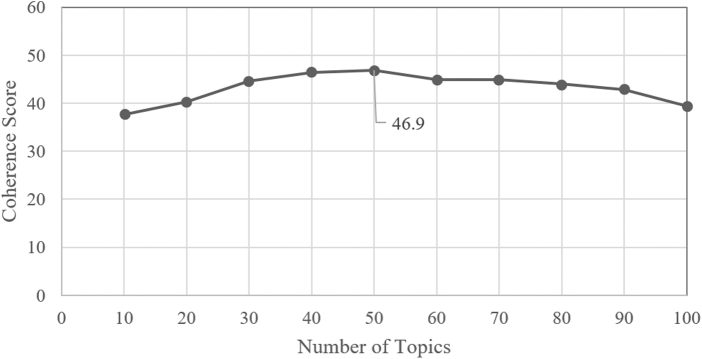
Coherence plot to determine optimal number of topics for Twitter LDA topic modeling. Fifty topics had the highest coherence score (46.9).

As shown in Figures 4 and 5, coherence scores were used to determine topics with the least variation between words, which resulted in 30 topics for Reddit and 50 topics for Twitter. Coherence plots are a technique that visually evaluates the quality of topic models by measuring semantic similarity between the top words in a topic. The *y*-axis represents the coherence scores, with higher scores indicating a more interpretable topic.

**FIG. 6.**
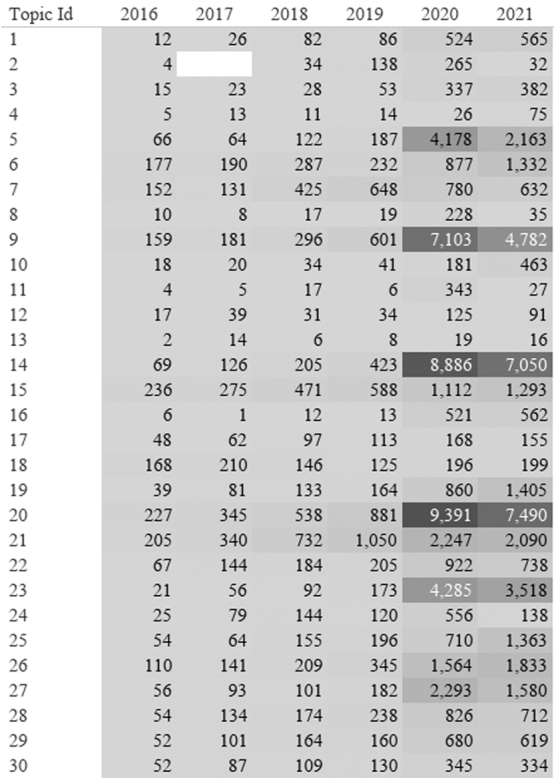
Heatmap of Reddit LDA topics from January 2016 through December 2021. Numbers represent the number of Reddit posts (submissions and comments) labeled in each topic by year. Dark *Gray* represents highest number and light *gray* represents lowest number.

Figure 6 shows the heatmap of topics in Reddit over time. The greatest concentration of posts can be found in 2020, coinciding with the onset of the pandemic.

The top topics presented in Table 4 represent Reddit's top five LDA topics using the relevance metric set to 1 (topic's words ranked based on probability within the topic) and 0 (topic's words ranked based on how much more probable they are in the topic compared with the entire corpus). After the onset of the pandemic (2020), the largest topic centered on words that appeared to discuss insurance, telehealth, and accessing doctors. The second and third most common topics included discussion of telehealth and therapy. The fourth most common topic included terms related to women's health, pain, and appointments. Finally, symptoms of COVID-19 and experiences related to the disease were discussed in topic 5.

**Table 4.** Top Five Reddit Latent Dirichlet Allocation Topics

Reddit LDA topic cluster results		
Topic	Most common topic terms (relevance metric = 1)	Most unique topic terms (relevance metric = 0)
5	covid, telehealth, symptoms, test, doctor, get, call, testing, days, go, day, tested, hospital, er, tests, also, like, care, appointment, said	shortness, fevers, albuterol, runny, wheezing, coughing, Mucinex, strep, sinus, scratchy, tonsillitis
9	telehealth, health, therapy, mental, services, online, therapist, help, also, clients, covid, may, via, therapists, sessions, person, state, free, insurance, find	codepad, Jodie, maglotti, psychologytody, Brenton, realdonaldtrump, surveycircle, innerscope, folxhealth, psypact
14	like, get, telehealth, feel, know, really, im, help, time, would, therapist, even, also, one, things, going, want, think, therapy, much	hurtful, transference, avoidant, grieve, stressfulafter, yaya, mom, jokingly, dissociating, tranquilum, dissociative, hypomania
20	telehealth, people, get, would, like, work, need, see, know, think, even, one, doctor, go, also, going, want, time, could, doctors	ndis, billable, tdap, geha, logistically, sunburnt, followups, lynx, pypy, ucsc
23	pain, telehealth, im, doctor, get, appointment, like, weeks, back, also, got, day, would, first, ive, days, week, one, comments, months	what_are_your, riteia, het, rincr, uterus, iud, diflucan, thrush, macrobid, cysts

“Topic” corresponds to the identification number of the LDA topic cluster. “Most Common Topic Terms” are the top words found in the topic cluster. “Most Unique Topic Terms” are the words that are unique within the cluster.

LDA, Latent Dirichlet Allocation.

**FIG. 7.**
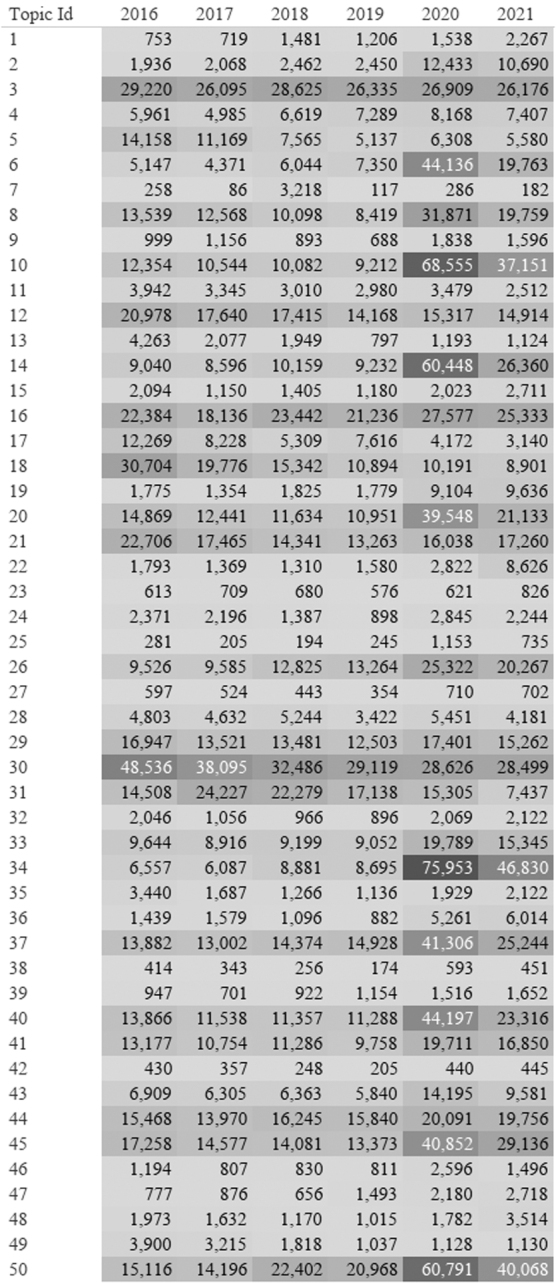
Heatmap of Twitter LDA topics from January 2016 through December 2021. Numbers represent the number of tweets labeled in each topic by year. *Dark Gray* represents highest number and *light gray* represents lowest number.

As seen in the heatmap in Figure 7, before the pandemic, Twitter showed a greater use of telehealth-related terms as compared with Reddit. Topic 30 focused on the term “digital health” and included discussion of businesses and investments; this topic was found before the pandemic, with the highest concentration in 2016. Twitter consistently used the terms “digital health” and “e-health,” while topic clusters with these terms were not impacted by the pandemic.

The most common theme (see Table 5 for the top Twitter themes) in the Twitter corpus related to using telehealth with making appointments (topic 34), including informal language about the difficulty making appointments. The second most common theme (topic 10) discussed the pandemic and adoption of new health care approaches. Therapy and mental health (topic 14) were discussed, including from businesses. Finally, the fifth most common topic cluster included discussion by specific business people whose names were removed from the unique topic terms to protect their identity.

**Table 5.** Top Five Twitter Latent Dirichlet Allocation Topics

Twitter LDA topic cluster results		
Topic	Most common topic terms	Most unique topic terms
10	covid, http, telehealth, pandemic, telemedicine, healthcare, nhttp, coronavirus, survey, care, new, use, virtualcare, adoption, health, increased, continue, post, response, patients	pandemic, amid, accelerated, widespread, respondents, amidst, ncov, uptick, skyrocketed, exponentially
14	telehealth, need, http, help, health, know, mental, therapy, please, support, people, us, care, make, get, resources, options, physical, clients, find	clients, therapists, ot, physicaltherapy, aptatweets, cal, patientchat, constituent, sliding, ots
30	digitalhealth, http, market, digital, healthcare, top, health, global, report, trends, companies, via, growth, industry, new, future, technologies, innovation, year, startups	market, invested, deals, forecast, cos, players, rock_heatlh, cagr, usd, accenture, trends
34	telehealth, get, go, back, appointment, time, got, going, doctor, can, see, one, last, call, like, telemedicine, today, still	got, appt, gonna, fucking, me, lol, wanna, gotta, idk, sucks
50	like, would, think, people, one, really, much, many, things, right, know, good, could, see, that, change, need, way, make, lot	direction, obvious, solved, unintended, quo, argument, cashless, shocking

“Topic” corresponds to the identification number of the LDA topic cluster. “Most Common Topic Terms” are the top words found in the topic cluster. “Most Unique Topic Terms” are the words that are unique within the cluster.

## Discussion

This study found that references of telehealth-related terms increased precipitously among Reddit and Twitter users after the start of the COVID-19 pandemic. While Twitter discussed telehealth-related topics before the pandemic, Reddit had minimal discussion around telehealth. Reddit's telehealth-related posts were predominately written after the pandemic started, with 84% of the posts about telehealth written after 2020. In comparison, Twitter had just under 50% of telehealth-related posts written after 2020.

The discussion of telehealth by both platforms tapered off as the pandemic progressed; however, Reddit users had a renewed interest in telehealth starting in September 2021. This study also found that the most commonly used telehealth-related terms were “telehealth” and “telemedicine.” Other terms such as “virtual health,” “virtual medicine,” and “digital health” were used less frequently on both platforms. The term “digital health” was, depending on the time frame, the third most common term on Twitter. The most common telehealth-related themes were insurance, mental health, and pain management on Reddit, and telehealth, pandemic-related businesses, and pandemic-related entrepreneurships on Twitter.

Previous research has shown the power of social media in monitoring news cycles. Del Gobbo et al. found that Twitter could be used to understand the themes discussed during the British exit from the European Union political debate.^30^ Specific to telehealth and Twitter, Pollack et al., found that discussion of telehealth spiked with the COVID-19 pandemic, and the tweets indicated that telehealth was being discussed favorably.^21^

A study looking at half a year of tweets found that there was an increase in tweets with the advent of the COVID-19 pandemic and that tweets were predominately created by entities promoting telehealth, such as vendors.^20^ Massaad's analysis studying a week of tweets during the pandemic found that the word “telehealth” was related to the words “covid,” “health,” “care,” “services,” “patients,” and “pandemic.”^19^

Reddit has not been as well characterized during the COVID-19 pandemic. Although Reddit has not been used to study telehealth, some studies have assessed specific subcommunities in Reddit such as opioid use, foster families, and COVID-19 long-haulers.^31–34^ These studies found that Reddit is a forum that provides users with a community to access support and information. Our results reiterated this point, with most of the “telehealth” and “COVID-19” discussions focused on using telehealth as a tool to remedy issues with mental health or relationships.

Lai et al. looked at one subreddit, “Ask Me Anything,” that allows Reddit users to ask subject matter experts about issues in their field.^35^ Their research found that users asked health experts mostly about symptoms, treatment, prevention, public health, and family. Anderson et al.'s study noted that while Twitter provides insights into social media conversation, research is needed in other platforms, such as Reddit, to understand how the pandemic affected telehealth.^22^

It is important to note that this analysis relied on subreddits to help understand why a Redditor was discussing telehealth-related terms. Unlike Twitter's hashtags, subreddits are topic-labeled communities and while a Redditor posts in that community, they are not tagging the topic of the post. The use of subreddits as a proxy for understanding the intent of the Redditor's post is common with subreddits used to narrow to the research topic of interest.^36–40^ This may be in part due to the smaller corpus obtained from a single subreddit rather than searching all of Reddit through time.

The themes presented in this research reveal that both Twitter and Reddit are used to understand telehealth but that the two platforms are used slightly differently. Twitter posts center on sharing information about current telehealth news, products, and services. The posts are often informational or advertising the potential of telehealth. Reddit is also used to share information and advertise but has an important subset of users that appear to rely on its pseudo-anonymous environment to ask for advice about mental health and physical health.

Both platforms are ideal for learning about general themes relating to telehealth and, especially, its strong ties to mental health, but Reddit provides more in-depth information on how telehealth can be used to solve health issues. This may be due to the much larger character limit in Reddit as compared with Twitter (Reddit allows 40,000 characters for a submission and 10,000 characters for a comment whereas Twitter only allows 280 characters).

This narrative format provides Redditors with a pseudoanonymous platform that can be ideal for providing first-person experiences as seen in its use in mental health research.^41–43^ Although not specific to telehealth, previous research has shown that social media and Reddit can be a “passive sensor” of mental health providing forecasting of mental health consultations.^43^ This may be indicative of Redditor's willingness to share health experiences in narratives submitted to Reddit.

The business-related tweets found on Twitter may be due to bots discussing products or trends with potentially 30% of tweets generated by bots.^44^ In addition, a Twitter sponsored survey found that almost 90% of Twitter users use the platform to find products and 76% have conversations on Twitter that lead to purchases.^45^ These Twitter-led insights may provide context on why so many business-related tweets were found in this telehealth corpus.

## Limitations and Future Research

Several limitations exist with this study. Human language is not always explicit, and tweets or posts may discuss telehealth or COVID implicitly. These posts would not be captured by our analysis, as we queried the Twitter and Reddit APIs using search terms (string matches). In addition, these data were collected using keyword searches and may have missed posts that misspelled “telehealth” or “telemedicine.”

Second, when comparing Twitter and Reddit, it is important to note the expectation of anonymity. Reddit provides users with a pseudonymous environment where posters can choose how much to divulge about themselves, sometimes maintaining complete anonymity. In comparison, Twitter exists in an environment that often relies on users providing their real names as exemplified by the Twitter blue checkmark that verifies a user's real identity.

Our findings that users were more likely to use Reddit to learn how to use telehealth or to talk about their medical experiences may be a result of the anonymous nature of Reddit. Third, it is also possible that the use of telehealth-related terms was not indicative of a post that was describing the health care field of telehealth. Fourth, LDA is a model that only allows one topic per post, meaning that a post that describes two or more topics has to be placed in just one topic.

This problematically may skew the percent each cluster covers.^46^ It is also possible that the LDA topic label was not correct for the post, which is discussed more thoroughly later about future research. The LDA may also be difficult to interpret from a human readability perspective: the word co-occurrence the algorithm finds may include terms that are seen as irrelevant or confusing. While our research includes telehealth domain experts, we may not have interpreted the topic clusters correctly. Fifth, users of social media often represent a younger, computer-literate demographic, which is not representative of the diversity of the United States.

Specifically, according to a 2021 Pew survey, just below half of Twitter users are ages 18–29 while only 36% of Reddit users are ages 18–29.^4^ Twitter users are 22% White, 29% Black, and 23% Hispanic while Reddit users are 17% White, 17% Black, and 14% Hispanic. These results should not be generalized to the full United States population. Finally, Twitter announced that they will no longer support the Academic API as a free resource.^47^

Changes to the API and the transparency around access to Twitter could impact future social media research and reproducibility of past work. Reddit's API is maintained by an outside entity and is dedicated to archiving the platform for researchers.^23^ The use of cross-platform analysis may be integral to continuing the field of social media analysis as current platforms' use policies change or platforms are removed.

Future research should cement the themes found in social media and telehealth by manually codifying Twitter tweets and Reddit posts into topics that can be used to create an annotated dataset. This dataset can be used for the creation of a machine that will identify individual posts across the dataset and will allow for multiple labels on one post (which LDA does not allow).

These codes could be studied over time to see if the pandemic had changed how users discussed telehealth before and after the pandemic. Our study stopped data collection in 2021. Future research may want to include more recent data to understand if the themes outlined in this article continue or if new topic areas are introduced.

In addition, the manual creation of labels could be compared with LDA to see if the LDA clusters had a similar distribution of themes. Past research often uses Twitter for the textual corpus; however, this study indicates that Reddit provides more information-rich context around health experiences of its users. Reddit may contain more patient experiences than Twitter, and further research exploring the quality of posts in this social media website could expand the field.

This study may help provide researchers and policymakers with insights into the use of Reddit and Twitter in the context of telehealth-related discussion. Our hope is that insights can be derived to support the use of social media to facilitate the use of telehealth for health care needs, particularly psychotherapy and counseling needs.

Future research may be able to build upon the themes and insights found in this work to directly impact supporting use of telehealth. Our study found that Reddit may be a more appropriate platform to discuss using telehealth or supporting forums to help Redditors understand access to telehealth especially as a tool to support mental health.

## Conclusion

Twitter and Reddit had extensive growth in the use of telehealth-related terms after the COVID-19 pandemic. While that growth has declined, telehealth-related terms are being used at a higher rate than before the pandemic. Users on both platforms used “telehealth” more than any other telehealth-related terms. The Twitter hashtag #covid was used in relation to #telehealth throughout the Twitter corpus. Twitter themes focused on telehealth appointments, telehealth increases due to the pandemic, and mental health.

Reddit showed themes connecting COVID-19 to telehealth, especially in reference to therapy and counseling. This study also showed that Reddit is an additional forum for understanding how users are discussing and using telehealth and that Reddit posts provide more nuanced information as compared with Twitter, which can be skewed more toward business-related tweets.

## Trial Registration

Not applicable, because this article does not contain any clinical trials.

## Abbreviations Used

API: Application Programming Interface
JSON: JavaScript Object Notation
LDA: Latent Dirichlet Allocation
